# Cardiac rehabilitation outcomes following a 6-week program of PCI and CABG Patients

**DOI:** 10.3389/fphys.2013.00302

**Published:** 2013-10-30

**Authors:** Herbert F. Jelinek, Zhaoqi Q. Huang, Ahsan H. Khandoker, Dennis Chang, Hosen Kiat

**Affiliations:** ^1^Australian School of Advanced Medicine, Macquarie UniversitySydney, NSW, Australia; ^2^Department of Biomedical Engineering, Khalifa University of Science, Technology and ResearchAbu Dhabi, United Arab Emirates; ^3^School of Community Health, Charles Sturt UniversityAlbury, NSW, Australia; ^4^Department of Cardiology, The Third Affiliated Hospital of Guangzhou Medical CollegeGuangzhou, China; ^5^Department of Cardiac Rehabilitation, Sydney Adventist HospitalSydney, NSW, Australia; ^6^Centre for Complementary Medicine Research, University of Western SydneySydney, NSW, Australia

**Keywords:** cardiac rehabilitation, exercise, percutaneous coronary angioplasty, coronary artery bypass drafting, heart rate variability

## Abstract

Coronary artery events requiring intervention are associated with depressed cardiac autonomic function. Whether a 6-week cardiac rehabilitation (CR) differs in effectiveness in improving exercise capacity (6MWT), cardiorespiratory function (peakVO_2_), and autonomic function (HRV) following either cardiac bypass surgery (CABG) or percutaneous coronary revascularization (PCI) is unknown. The current study therefore compared the change in 6MWT and peak VO2 to HRV variables following a 6-week CR program and with patients having either PCI or CABG. Thirty-eight patients, (PCI, *n* = 22 and CABG, *n* = 16) participated in the CR program and results for pre and post 6 min walk test (6MWT), peakVO_2_, and heart rate variability (HRV) were obtained. Our study has shown that a 6 weeks program following either PCI or CABG improves function. However, the effect on post-CABG differs to that of post-PCI patients. The change in distance walked (6MWT, metres) was higher in the CABG (Δ6MWT: 61, *p* < 0.001) compared to the PCI group (Δ6MWT: 41, *p* < 0.001). Maximum exercise capacity (peak VO_2_, ml/kg.min) also changed significantly with a greater change in the CABG group (ΔPCI: 0.7, *p* < 0.001; ΔCABG: 1.0, *p* < 0.001) but did not reach normal population values. Although an improvement in HRV parameters was noted for the PCI group, a statistically significant improvement in HRV was observed only in the CABG group for the following; SDNN (ms) (baseline vs. post-rehabilitation (median ± IQR): 31.2 ± 25.6 vs. 51.8 ± 23.1, *p* < 0.01), RMSSD (19.32 ± 19.9 vs. 42.1 ± 34.2, *p* < 0.01); LF (ms^2^) (191 ± 216 vs. 631 ± 693, *p* < 0.01) and HF (107 ± 201 vs. 449 ± 795.0, *p* < 0.05). A significant interaction in the PCI group but not in the CABG group was observed using correlation analysis between the 6MWT and peak VO_2_ with HRV parameters indicating that being healthier that is, a better 6MWT and peak VO_2_ led to better HRV results but no significant effect of CR in the PCI group. When the results were investigated for baseline 6MWT and peak VO_2_ effect using a covariate analysis, a significant influence of CR on HRV parameters was retained in the CABG group (*p* = 0.0072). Our study indicates that a 6-weeks CR program benefits both patient groups in terms of exercise capacity, cardiorespiratory function and autonomic nervous system modulation of heart rate, with CABG patients showing the most improvement. HRV can be a useful additional variable to gauge cardiac function following CR.

## Introduction

Percutaneous coronary angioplasty intervention (PCI) and coronary artery bypass grafting (CABG) are effective and established treatments for clinically significant coronary artery disease (CAD) (Eagle et al., 2004; Bravata et al., 2007). The long-term survival benefits of cardiac rehabilitation (CR) among patients with CAD have been documented following several large-scale trials and meta-analyses with no direct comparisons between cardiac intervention (CABG vs. PCI) or current measures, including the 6-min walk test and peak oxygen volume (6MWT, peak VO_2_) (Giannuzzi et al., 2003; Taylor et al., 2004; Leon et al., 2005; Suaya et al., 2009). In recent papers there has been some speculation this may be due to an improvement in cardiac autonomic function (Lucini et al., 2002; Routledge et al., 2010).

The 6MWT is now used routinely to demonstrate the physical and physiological benefits of CR following coronary intervention with peak VO_2_ still used in major metropolitan hospitals (Fiorina et al., 2007). Recently Soumagne reported improvement in functional capacity following CR but did not report direct comparisons between PCI and CABG (Soumagne, 2012).

Heart rate variability (HRV) is a valid marker of cardiac autonomic activity and complexity that reflects sympathetic and parasympathetic balance and overall tone (Mäkikallio et al., 2006; Jelinek et al., 2010; McLachlan et al., 2010). HRV can be measured in both time and frequency domains, where global activity in the time domain is indicated by the standard deviation of the RR intervals (SDNN) and parasympathetic function by the root mean square of the standard deviation of the RR intervals (RMSSD) (TFESC, 1996). Sympathetic output in the frequency domain is loosely correlated with low frequency (LF) power, although a parasympathetic component has been noted, while parasympathetic/vagal output is in part correlated with high frequency (HF) power of the HRV spectrum (Grassi et al., 2009). The LF/HF ratio is a derived value from the calculated HF and LF spectral component (Reed et al., 2005; Billman, 2013). Reduced HRV may be associated with abnormal adaptability of the cardiac autonomic nervous system to changes in cardiac pathology and may increase risk of adverse or fatal cardiac events (Kleiger et al., 1987; Quintana et al., 1997; Weber et al., 1999; Jelinek et al., 2010).

Parasympathetic indices are reduced in patients within 24 h after PCI (Tseng et al., 1996; Osterhues et al., 1998; Wennerblom et al., 2000; Kanadasi et al., 2002). However, this reduction in parasympathetic function appears to be a transient phenomenon (Osterhues et al., 1998; Kanadasi et al., 2002; Janowska-Kulińska et al., 2009). In patients with greater than one target-vessel affected and/or with other comorbidities, HRV remains lower due to several factors (Tseng et al., 1996; Birand et al., 1998; Wennerblom et al., 2000; Kanadasi et al., 2002). In advanced heart failure with low left ventricular ejection fraction autonomic dysfunction may be due to a decrease in muscarinic receptor density, or changes in neuro-hormonal output leading to a decrease in parasympathetic output and an increase in sympathetic activity (Eckberg et al., 1971). In further studies, HRV has been shown to be independent of ejection fraction, extent of CAD and other variables, where a decreased HRV is a potent independent predictor of mortality in the 12 months following elective coronary angiography in patients without recent myocardial infarction (Rich et al., 1988). A number of studies have found that CR can improve both HRV and exercise capacity in patients following PCI (Chien et al., 2006; Munk et al., 2009; Baumert et al., 2011).

In general, outcomes for post-CABG reported in earlier studies differ to PCI in that impairment of cardiac autonomic regulation assessed by HRV remains suboptimal for several months or years post CABG and an increased risk of atrial fibrillation remains present (Demirel et al., 2002; Bauernschmitt et al., 2004; Cygankiewicz et al., 2004; Wu et al., 2005; Laitio et al., 2006; Kalisnik et al., 2007). Accordingly, strategies resulting in favorable recovery of cardiac autonomic tone as soon as possible after CABG may be clinically important in these patients. Long-term outpatient exercise-based CR has been reported to positively improve exercise capacity and cardiac autonomic function post-CABG hospital discharge in programs of 2 months duration or longer (Hirschhorn et al., 2008; Baumert et al., 2011). However, data evaluating the impact of CR on HRV in outpatients after CABG are limited (Iellamo et al., 2000; Baumert et al., 2011). It is uncertain whether short-term CR applied post-CABG during an outpatient CR program has a substantial beneficial impact on HRV and how this compares to current measures of exercise capacity (6MWT) and cardiorespiratory function (peak VO_2_). No study has directly compared the physiological parameters measured as 6MWT and peak VO_2_ and cardiac autonomic function improvements for CABG and PCI patients following a 6-week CR program.

The current study prospectively compared the impact of a 6 weeks CR program on baseline HRV parameters and exercise capacity outcomes with patients recovering from both PCI and CABG in a standardized program.

## Methods

### Participants

The project was conducted in accordance with ethics guidelines and approval by the Sydney Adventist Hospital and Charles Sturt University Human Ethics Research Committee. All participants were provided with an information sheet and gave informed consent.

The participants who agreed to participate in this study were prospectively and consecutively enrolled between July and December 2010 and part of the CR program at the Sydney Adventist Hospital, Wahroonga, NSW, Australia. All patients had revascularization procedures (either PCI or CABG) within 1 month of enrolment. Patients enrolled into CR were excluded from the study if the principal diagnosis was of cardiac failure, valvular surgery, or patients who had a documented myocardial infarction within 6 months prior to study enrolment. Participants were also excluded from the analysis if they were unable to complete the 6-week CR program or could not participate in the designated exercise program.

### Rehabilitation program

Exercise sessions were performed three times per week for 6 weeks at 55–70% of peak VO_2_, measured during the first exercise session at commencement of the CR program combined with a patient perceived exertion rating of 11–13 (fairly light to somewhat hard) on the Borg scale (Borg, 1982). The program included 16 periods of exercise training and six education sessions on cardiovascular risk factors, life style modification measures and the pathophysiology of atherosclerosis. The exercise component of the program was conducted according to the National Heart Foundation of Australia & Australian Cardiac Rehabilitation Association (NHFA and ACRA, 2004) guidelines. Each participant was given an individualized exercise program consisting of aerobic exercise (cycle ergometry, treadmill walking, and rowing) that was devised by an exercise physiologist to ensure the participants could exercise continually throughout the session at the prescribed level of intensity. Each session consisted of stretching and warm-up exercise (5–10 min), endurance training (15–20 min), resistance training (10–15 min), or strength training (10–15 min) and cool-down/relaxation exercise (5–10 min). Participants were also advised to complete a home walking program, as recommended by the National Heart Foundation to achieve 30 min of moderate intensity physical activity on most or all days of the week.

### Data collection

The 6-min walk test (6MWT) is a standard measure of functional walking capacity and as such provides insight into the likely effect of participation on patients' ability to carry out activities of daily living (Fiorina et al., 2007). This test consists of walking up and down an 18 m indoor track as many times as possible within a 6-min period (NHFA and ACRA, 2004; West et al., 2012). In accordance with the American Thoracic Society guidelines this test was conducted twice prior to commencing CR, with the better of the two tests recorded as baseline (Argyropoulos and Harper, 2002). Patients were allowed to rest if required, and the assessment was ceased if there was angina pectoris or undue shortness of breath, which would normally limit activity and/or necessitate the use of coronary vasodilator therapy. Patients were advised of elapsed time at each minute of the assessment. No other encouragement was given. The peak VO_2_ was collected throughout the 6MWT and calculated using the American College of Sports Medicine formula (ACSM, 2006).

A 20-min, 3-lead ECG recording (PowerLab 4/30, LabChart Version 7, Castle Hill, NSW, Australia) was obtained prior and post CR. Time series variables were analyzed over the 20-min recording range, whereas frequency domain measures were analyzed using the accepted 5-min successive interval method to avoid the influence of non-stationarity (Task Force, 1996). The time domain variables considered in this study were the mean RR interval and its standard deviation of the N-N intervals (SDNN), representing the overall HRV and its root mean square successive difference (rMSSD), representing the vagal tone. The frequency-domain variables measured were LF and HF and the ratio LF/HF, which provide information on vagal and sympathetic input modulating HRV (Carney et al., 2005).

Other information obtained from patients attending the CR included medications, blood pressure, smoking, and diabetes status. Height and weight were measured, and the body mass index (BMI) calculated. Waist circumference was measured within an accuracy of 0.1 cm. Blood pressure was measured with a clinical zero sphygmomanometer. The average of two measurements was used to determine blood pressure. A cardiac history questionnaire was given to all participants to ascertain any other factors that may impact on their exercise capacity and HRV.

### Data analysis

All the data were analyzed by LabChart version 7 (ADInstruments, Castle Hill, Sydney, Australia). In accordance with the American Thoracic Society guidelines (ATS, 2002), 6MWT results were expressed as an absolute value. Exercise capacity was expressed as peak VO_2_ in ml/kg.min.

Statistical analysis was performed using SPSS Version 20 (Copyright IBM Inc.). The Wilcoxon Signed-Ranks Test was used to compare HRV parameters for the CABG and PCI groups before and following CR. A student *t*-test was used to compare the 6MWT and peak VO_2_ results before and following CR. To assess the correlation on a nominal scale the Pearson's correlation test was used. Covariate analysis to model the influence of 6MWT and peak VO_2_ on effectiveness of CR was determined using Friedman's test. Differences were considered significant when *p* < 0.05. Values were expressed as means and standard deviation for normally distributed data and medians and interquartile range (IQR) if the data was not normally distributed.

## Results

### Baseline values of patients

Forty-two patients were consecutively enrolled after successful cardiac intervention procedures if they agree to the research and provided informed consent. The PCI group consisted of 25 patients and the CABG group of 17 patients. One patient was unable to complete the CR program, two missed the follow-up appointment and one patient was hospitalized during the rehabilitation program. No patients experienced angina during the exercise component of the rehabilitation program. Data from 22 patients in the PCI group and 16 patients in the CABG group were used for the final analysis.

There were no significant differences in risk profile including age, blood pressure, smoking, and diabetes, and clinical presentation between the two groups (Table 1). Medication use was similar between the two groups apart from a significant difference in the PCI group used angiotensin converting enzyme inhibitors (ACEI). One patient in the PCI group and two patients in the CABG group discontinued β-blocker therapy and were excluded from the analysis.

**Table 1:** **Patient demographics and clinical history**.

	**PCI (%)**	**CABG (%)**	**P**
**BASELINE CHARACTERISTICS**			
Male/female (%)	18 (81.8)/4 (18.2)	13 (81.3)/3 (18.7)	NS
Age (years)^#^	62.5 ± 9.9	64.9 ± 8.8	NS
BMI (kg/m^2^)	27.5 ± 3.9	27.3 ± 4.6	NS
Waist circumference (cm)	98.1 ± 10.3	97.5 ± 12.0	NS
**HISTORY**			
Past cardiac surgery (%)	1 (4.5)	3 (18.8)	NS
Myocardial infarction (%)	11 (50.0)	5 (31.3)	NS
Hypertension (treated) (%)	8 (36.4)	10 (62.5)	NS
Valvular disease (%)	0 (0.0)	3 (18.8)	NS
Arrhythmia (%) AF^**^	1 (4.5)	3 (18.8)	NS
Diabetes (%)	5 (22.7)	1 (6.3)	NS
Hyperlipidaemia (%)	17 (77.2)	14 (87.5)	NS
Stroke (%)	2 (9.1)	1 (6.3)	NS
Kidney problem (%)	1 (4.5)	1 (6.3)	NS
Family history (%)	14 (63.6)	15 (93.8)	NS
Smoker/ex smokers (%)	1 (4.5)/8 (36.4)	0 (0.0)/3 (18.8)	NS
Depression (%)	6 (27.3)	4 (25.0)	NS
**MEDICATION**			
β-blockers/Stopped (%)	11 (50.0)/1 (4.5)	8 (50.0)/2 (12.5)	NS
Calcium antagonists (%)	0 (0.0)	1 (6.3)	NS
Diuretics (%)	2 (9.1)	2 (12.5)	NS
Angiotensin converting enzyme inhibitors	14 (63.6)	4 (25.0)	0.025^*^
Antiplatelet treatment/bleeding	22 (100.0)/1 (4.5)	16 (100.0)/0 (0.0)	NS

# Mean and standard deviation;

* PCI vs. CABG p < 0.05;

** Atrial fibrillation.

### Clinical variables

BMI and waist circumference were measured before and after CR. No significant differences were observed after the 6-week CR programme (Table 2).

**Table 2:** **BMI and waist circumference in the CABG and PCI groups**.

**Variables**	**Baseline (mean ± SD)**	**6 weeks (mean ± SD)**	***P*-value**
PCI_BMI (kg/m^2^)	27.5 ± 4	27.1 ± 4	0.17
PCI_Waist^**^ (cm)	98.1 ± 10	96.6 ± 9	0.09
CABG_BMI (kg/m^2^)	27.3 ± 4	27.3 ± 4	0.89
CABG_Waist (cm)	97.5 ± 12	96.5 ± 12	0.12

** waist circumference.

### Heart rate variability

Significant increases were seen for SDNN, RMSSD, LF, and HF in the CABG group following CR compared to baseline (Table 3).

**Table 3:** **Changes of the HRV indices in PCI and CABG group**.

**Variables**	**Baseline (median ± IQR)**	**6 weeks (median ± IQR)**	***P*-value**
**PCI**			
SDNN (ms)	49.1 ± 45.3	53.42 ± 35.36	NS
rMSSD	30.17 ± 46.1	35.68 ± 38.5	NS
LF (ms^2^)	438 ± 1414	781 ± 1389	NS
HF (ms^2^)	291 ± 1309	376 ± 821	NS
LF/HF	1.47 ± 0.9	1.87 ± 1.7	NS
**CABG**			
SDNN (ms)	31.19 ± 25.6	51.8 ± 23.1	0.005
rMSSD	19.32 ± 34	61.6 ± 54	0.02
LF (ms^2^)	191 ± 216	631 ± 639	0.001
HF (ms^2^)	106 ± 201	449 ± 795	0.02
LF/HF	1.09 ± 1.14	1.06 ± 2.01	NS

There was no significant change in HRV post CR in the PCI group for all HRV measures. Significant differences were seen for the majority of HRV measures for the CABG group when comparing post CR to baseline levels (Table 3).

Analysis of the difference in the extent of change (Δ) between the CABG and PCI groups for the time and frequency domain parameters following CR revealed no significant differences in the HRV parameters. However, CABG patients showed a greater improvement in all HRV measures.

A significant interaction in the PCI group but not in the CABG group was observed using covariant analysis between the 6MWT and peak VO_2_ with HRV parameters, indicating that being healthier that is, better 6MWT and peak VO_2_, led to better HRV results but no significant effect of CR in the PCI group. When the results were investigated for baseline 6MWT and peak VO_2_ in the CABG group a significant influence of CR on HRV parameters was retained in the CABG group (*p* = 0.0072).

### Exercise capacity

Clinically significant improvements in exercise capacity were observed in both PCI and CABG groups following CR as judged by the American Thoracic Society guidelines (2002), which stipulate that an increase of 54 m in the 6MWT is the minimum distance required (Table 4).

**Table 4:** **6MWT distance and metabolic variables in both groups at baseline and after 6 weeks**.

**Variables**	**Baseline**	**6 weeks**	***P*-value**
PCI_6MWT Distance (m)	548.1 ± 62.0^*^	589.0 ± 78.1	0.001
PCI_Peak VO_2_ (ml/kg.min)	12.6 ± 1.0	13.3 ± 1.3	0.001
CABG_6MWT Distance(m)	504.3 ± 93.5	565.8 ± 98.8	0.001
CABG_Peak VO_2_ (ml/kg.min)	11.9 ± 1.6	12.9 ± 1.6	0.001

* Mean and standard deviation.

Peak VO_2_ increased significantly in both groups. However, it remained below international and Australian population norms that are set between 20 and 35 ml/kg.min (see Table 4). CABG patients improved by 8% and PCI by 1% compared to baseline following the CR program.

## Discussion

Our study is the first to investigate whether there is a difference in CR outcomes following a 6 week, moderate-intensity exercise program for patients that have undergone either CABG or PCI with differing levels of parasympathetic suppression at baseline (within the first month following intervention but immediately prior to rehabilitation). Effect of CR on HRV was compared to the traditional measures of 6MWT and peak VO_2_. CABG patients clearly entered the program with lower HRV parameters, particularly for LF power activity and total HRV, which may be due to more extensive myocardial or cardiovascular disease and the more invasive intervention (Santangeli et al., 2008; Lakusic et al., 2009). CABG intervention has been shown to decrease HRV post-CABG intervention with HRV parameters not improving past preoperative levels even after 6 months (Kuo et al., 1999). There were two principal findings: (1) CR significantly improved both HF power and LF power in the CABG group, which suggests significant changes to the extent or functionality of the autonomic nervous system, (2) CR had a significant impact on the exercise capacity in both groups. Our study also supports that a short 6-week CR program as recommended by the Australian guidelines is sufficient to improve exercise capacity, cardiorespiratory function regardless of intervention (PCI or CABG) and cardiac autonomic function in CABG patients (NHFA and ACRA, 2004). Our study agrees with previous reports that despite patients with diabetes having a higher risk of adverse cardiac events, adherence to a CR program can improve functional capacity (Hindman et al., 2005).

Time after intervention is an important component that influences CR outcomes. We recruited patients between 2 and 4 weeks after intervention and observed that the baseline values for HRV in the CABG group were much lower than for the PCI group in agreement with previous studies (Demirel et al., 2002; Cygankiewicz et al., 2004; Laitio et al., 2006). While there was a correlation between HRV parameters and 6MWT as well as peak VO_2_, this did not explain all the variance, hence indicating HRV is an independent physiological measure that provides information on the outcome of CR. HRV may be a more sensitive marker for effectiveness of improvement in cardiac function. HRV can be determined from 2-, 5-min, or longer lead II ECG recordings (Buchheit et al., 2007). 6MWT did show an improvement in both groups with only the CABG group meeting the recommended 56 meters cut-off compared to the PCI group. Peak VO_2_, demonstrated significant improvement as well for both groups, but results remained well below published population norms for our study and age range. This lower than previously reported outcome in peak VO_2_ may be due to higher baseline values on entry to the program especially in the PCI group or possibly due to patient motivation, home-based compliance, age, gender, and fitness of the patients. However, even modest improvements are correlated with better long-term outcomes in cardiorespiratory function (Swank et al., 2012).

CR has been demonstrated to decrease mortality following PCI (Goel et al., 2011) and either improved survival or morbidity compared to no CR following CABG in longitudinal studies (Hedbäck et al., 2001). However, comparative studies addressing CR effectiveness with respect to the type of cardiac intervention (PCI vs. CABG) and improvement in HRV compared to the traditional measures of 6MWT and peak VO_2_ has not been investigated in the one study design. Our study indicates that CR following CABG may improve long-term outcome by reducing the risk of future sudden cardiac death by increasing parasympathetic tone and therefore HRV (Peng et al., 1995).

The effectiveness of CR is influenced by type of surgery and length of rehabilitation program (Eagle et al., 2004). CABG patients usually have more than one target vessel involved, post-operative pain, morbidity, and hence their recovery period is often longer. Patients undergoing PCI, on the other hand, can begin CR 24–48 h after PCI if there is no evidence of hematoma at the catheterization site (West et al., 2012). Length of CR may be a factor in its effectiveness, with many studies reporting an 8-week period (Austin et al., 2004; Freyssin et al., 2012).

Our study shows in a single center cohort that CR consistently improved HRV in patients following CABG even when pre CR values of 6MWT and peak VO_2_ are considered.

## Conclusion

This study indicates that the effect of CR is of benefit to patients with reduced parasympathetic tone prior to the start of CR and that CR has a greater effect in post-CABG compared to post-PCI. In addition HRV is independent of 6MWT and peakVO2, suggesting that HRV is a useful additional measure to employ for CR.

## Author contributions

Herbert F. Jelinek: Lead investigator, organized study protocol, data interpretation, statistical analysis, and writing; Zhaoqi Q. Huang: Cardiologist, performing all experimental work, data analysis, and writing; Hosen Kiat: Cardiologist in charge, responsible for developing exercise protocol for participants, study protocol, interpretation, and writing of article; Dennis Chang: Data interpretation and writing of article; Ahsan H. Khandoker: ECG analysis, data interpretation, and writing of article.

### Conflict of interest statement

The authors declare that the research was conducted in the absence of any commercial or financial relationships that could be construed as a potential conflict of interest.
